# Enhancement of Contralesional Motor Control Promotes Locomotor Recovery after Unilateral Brain Lesion

**DOI:** 10.1038/srep18784

**Published:** 2016-01-06

**Authors:** Xu-Yun Hua, Yan-Qun Qiu, Meng Wang, Mou-Xiong Zheng, Tie Li, Yun-Dong Shen, Su Jiang, Jian-Guang Xu, Yu-Dong Gu, JoeZ. Tsien, Wen-Dong Xu

**Affiliations:** 1 Department of Hand Surgery, Huashan Hospital, Shanghai Medical College, Fudan University, Shanghai, China; 2 Department of Hand and Upper Extremity Surgery, Jing’an District Central Hospital, Shanghai, China; 3 Hand-Foot Surgery Department, Shandong Provincial Hospital, Shandong, China; 4 Brain and Behavior Discovery Institute and Department of Neurology, Medical College of Georgia, Georgia Health Sciences University, Augusta, GA 30907, USA; 5 Yunnan BanNa Primate Model Research Center, BanNa Biomedical Research Institute, Xishuangbanna, Yunnan, China; 6 State Key Laboratory of Medical Neurobiology, Fudan University, Shanghai, China

## Abstract

There have been controversies on the contribution of contralesional hemispheric compensation to functional recovery of the upper extremity after a unilateral brain lesion. Some studies have demonstrated that contralesional hemispheric compensation may be an important recovery mechanism. However, in many cases where the hemispheric lesion is large, this form of compensation is relatively limited, potentially due to insufficient connections from the contralesional hemisphere to the paralyzed side. Here, we used a new procedure to increase the effect of contralesional hemispheric compensation by surgically crossing a peripheral nerve at the neck in rats, which may provide a substantial increase in connections between the contralesional hemisphere and the paralyzed limb. This surgical procedure, named cross-neck C7-C7 nerve transfer, involves cutting the C7 nerve on the healthy side and transferring it to the C7 nerve on the paretic side. Intracortical microstimulation, Micro-PET and histological analysis were employed to explore the cortical changes in contralesional hemisphere and to reveal its correlation with behavioral recovery. These results showed that the contralesional hemispheric compensation was markedly strengthened and significantly related to behavioral improvements. The findings also revealed a feasible and effective way to maximize the potential of one hemisphere in controlling both limbs.

Unilateral brain injury is an important cause of long-term functional disability. Brain plasticity is the main mechanism for functional recovery post injury, particularly in the chronic stage^1^. How to enhance this ability of self-recovery by maximizing appropriate brain plasticity has been a wide concern. Several mechanisms have been shown to be involved in this process of spontaneous recovery, either solely or synergistically. The ipsilateral pathway, which takes advantage of the contralesional hemisphere in controlling both hands, has great potential, although no consensus has currently been achieved^2^,^3^,^4^.

Some authors have reported that mild brain lesions, which preserve most of the contralateral (ipsilesional) cortico-motor projections, exhibit the most favorable functional recoveries, and in contrast, exclusive contralesional activation only produces disappointing functional recovery^5^,^6^. However, enhancement of contralesional activation is usually a concomitant result associated with larger brain lesions. Moreover, the ipsilateral fibers only account for 10–20% or even less of all corticospinal projections^7^. Thus, it is difficult to determine whether the contralesional activation is helpful or obstructive. Some neuroscientists have suggested that contralesional hemispheric activation might require re-enforcement to successfully compensate for impaired function after brain injury. However, until recently, no effective methods have emerged to directly enhance the contralesional motor control^8^. Thus, a novel surgical procedure is required to resolve this problem.

Our assumption is derived from the idea of peripheral nerve transfer, which is the process of sacrificing a relatively less important nerve in the normal upper extremity as a donor and transferring it to the appropriate recipient nerve on the affected side. Thus, a successful connection between the contralesional hemisphere and the affected upper extremity can be constructed. Nerve fibers in the brain and at the spinal level are complicated and are difficult to discern. The most feasible method is to operate on the neural pathway at the peripheral level.

Contralateral C7 nerve root transfer, which was first introduced by Gu^9^, has been used in the treatment of brachial plexus avulsion injuries (BPAI) with promising outcomes^10^,^11^. In this procedure, the seventh cervical nerve root of the healthy side was transferred to recipient nerves of the injured side, such as the median nerve. Clinical and experimental studies have shown that a contralateral C7 nerve root transfer does not cause damage to the function of the donor upper extremity, and the repaired extremity could be controlled by the motor cortex after appropriate cortical reorganization^12^,^13^,^14^,^15^.

In our present surgical design, 20% of the nerve fibers that innervate the intact upper extremity were severed and transferred to the paralyzed limb. Thus, the quantity of the connection between the contralesional hemisphere and the paralyzed limb could be significantly enhanced. Here, we described not only the encouraging findings obtained from behavioral observation but also neurophysiological and neuroimaging evidence that demonstrates the underlying recovery processes in an animal model.

## Results

### Behavioral observation

**Figure 1:**
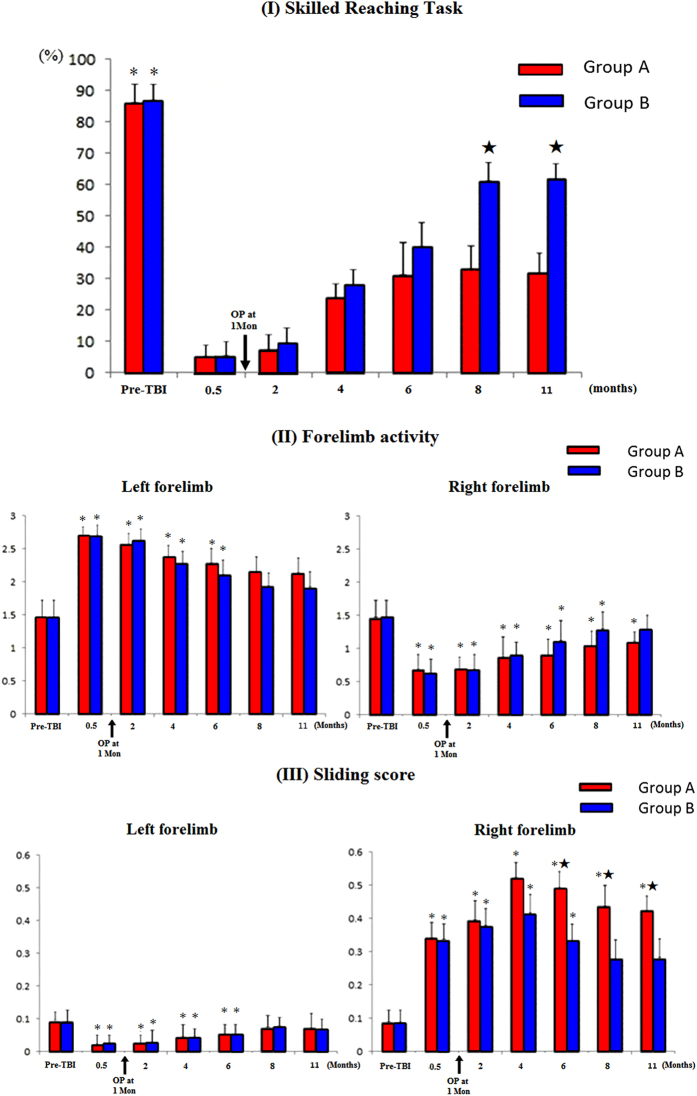
Scores of behavioral tests pre- and at different intervals after traumatic brain injury (TBI) over the left hemisphere. Group A (TBI_Control): All rats showed right forelimb paralysis immediately after TBI, with decreased skilled reaching task and forelimb activity (FLA) scores and significantly increased sliding scores. The behavioral function started to improve since 0.5 months after TBI and stopped improving from 4 months to 11 months. Scores of the behavioral tests were still significantly worse than baseline scores at 11 months. In addition, the use of the intact (left) forelimb increased immediately after TBI, with significantly lower FLA and higher sliding scores than the pre-TBI level. The left forelimb function returned to pre-TBI level at 8 months and then stabilized thereafter. Group B (TBI_C7-Transfer): In the first 4 months after TBI, the behavioral scores in Group B (TBI_C7-Transfer) had a similar tendency with that in group A (TBI_Control). However, the scores increased significantly at 6 months and then stabilized from 8 to 11 months. There were significant differences between two groups in the skilled reaching task from 8 to 11 months and in sliding score from 6 to 11 months. * indicating a significant difference between each interval after TBI and pre-TBI values; ★indicating significant difference between two groups

Lesions were made in the left motor cortex in two groups of rats, and only in Group B (TBI_C7-Transfer) was there was the contralateral C7 nerve root transfer. In the skill reaching task, group A (TBI_Control) and B (TBI_C7-Transfer) showed severe motor deficits in the right forelimbs immediately after brain ablation, but all animals showed spontaneous functional recovery by the end of 4 months. By the end of 8 months, the scores were stable, and successful reaching scores in groupA (TBI_Control) and B (TBI_C7-Transfer) achieved (33 ± 4)% and (61 ± 5)%, respectively, with a significant difference (*p* < 0.05) (Fig. 1).

In the Limb-use asymmetry test, after brain ablation, all of the rats preferred using their left forelimbs when they initiated rearing and exploring, with the injured right forelimbs sliding along the cylinder wall. The tests exhibited significant decreased forelimb activity (FLA) scores and increased sliding scores of the right forelimbs in group A (TBI_Control) and group B (TBI_C7-Transfer) at 0.5 month (Fig. 1).

This recovery tendency became stable at the end of 8 months. Group A (TBI_Control) could finish exploration with the right forelimbs, but they were still slower than with the left forelimbs. Moreover, group A (TBI_Control) still showed severe motor dysfunction of the right forelimbs. The sliding scores of the right forelimbs in group A (TBI_Control) were significantly higher than group B (TBI_C7-Transfer) (*p* < 0.05). The behavioral scores of the left forelimbs of rats in both group A (TBI_Control) and B (TBI_C7-Transfer) reached baseline levels by the end of 7 months (*p* > 0.05) (Fig. 1).

### Micro-PET Studies

To examine *in vivo* brain activity substrates underlying functional differences, we employed micro-PET to measure cerebral glucose metabolism in the rat sensorimotor cortex of 6 rats in group C (TBI_Control) and group D (TBI_C7-Transfer) and normal control animals.

**Figure 2:**
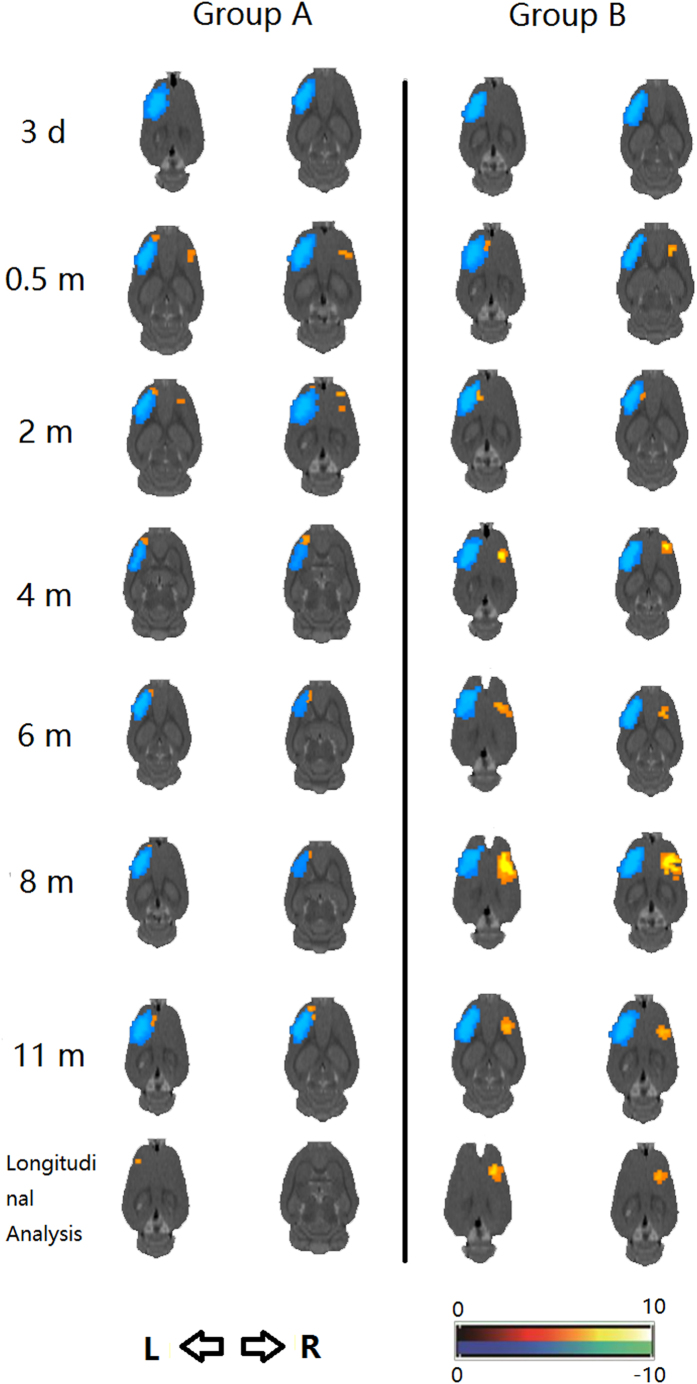
Micro-PET results of the paired t-test for comparison between group A (TBI_Control) and B (TBI_C7-Transfer) at different intervals after traumatic brain injury (TBI) over the left hemisphere and longitudinal analysis. Group A (TBI_Control): Significant decrease in glucose metabolism was detected in the left hemisphere at 3 days after TBI. Since 0.5 months, we observed significantly increased glucose metabolism in both the residual ipsilesional hemisphere and contralesional hemisphere. The residual ipsilesional hemisphere was gradually reduced but was constantly expressed from 0.5 months to 11 months, while the contralesional hypermetabolic area disappeared at 4 months. In the longitudinal analysis, the residual ipsilesional hemisphere was positively correlated with spontaneous functional recovery of the paralyzed forelimbs. Group B (TBI_C7-Transfer): Increased glucose metabolism in both the residual ipsilesional hemisphere and contralesional hemisphere at 0.5 months after TBI. However, the contralesional hypermetabolic area disappeared at 2 month after TBI (i.e., 1 month after the cross neck C7-C7 transfer), reappeared at 4 months and then improved and peaked at 8 months. However, it finally decreased at 11 months after TBI. In contrast to group A (TBI_Control), hypermetabolism in the residual ipsilesional hemisphere disappeared since 4 months after TBI and did not reoccur. In a longitudinal study, activation of the contralesional hemisphere was the main contributor to the behavioral recovery.

A significant decrease in cerebral glucose metabolism (CGM) was detected in the left sensorimotor cortex of group C (TBI_Control) rats at 3 days after traumatic brain injury (TBI) using micro-PET tests. At 0.5 month after TBI, we observed significantly increased CGM in both the residual ipsilesional hemisphere and contralesional hemisphere (Fig. 2, Fig. S1 in Supplementary Material). The hypermetabolism of the residual ipsilesional hemisphere gradually reduced but was still present from 0.5 month to 11 months. In the longitudinal analysis, the hypermetabolism of the residual ipsilesional hemisphere positively correlated with spontaneous functional recovery of the paralyzed forelimbs.

In group D (TBI_C7-Transfer) animals, micro-PET showed increased CGM in both the residual ipsilesional and contralesional hemisphere at 0.5 month after TBI. Critically, the hypermetabolic area in the contralesional hemisphere disappeared at 2 months after TBI (i.e., 1 month after cross neck C7-C7 transfer), reappeared at 4 months, and then peaked at 8 months after TBI (Fig. 2, Fig. S2 in Supplementary Material). In contrast to group C (TBI_Control), hypermetabolism in group D(TBI_C7-Transfer) in the residual ipsilesional hemisphere disappeared by 4 months. In the longitudinal study, the hypermetabolic area in the contralesional hemisphere was the main correlate to behavioral recovery.

### Intracortical Microstimulation

**Figure 3:**
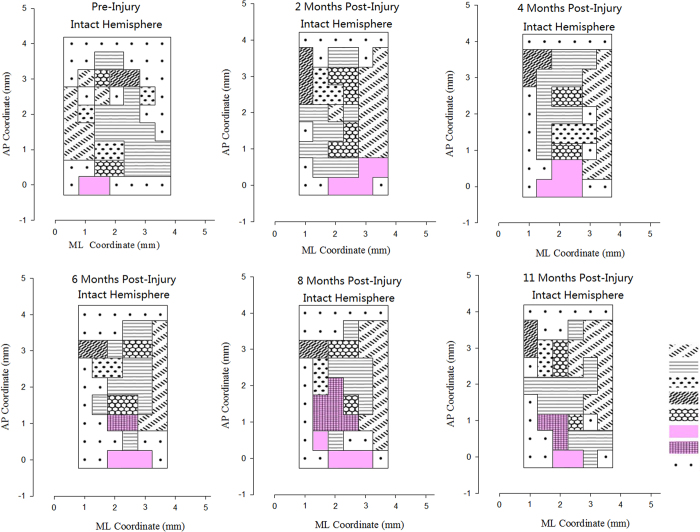
The primary motor cortex (M1) maps of intact hemispheres in group F (TBI_C7-Transfer) from 2 to 11 months after cross neck C7-C7 nerve transfer. At 6th month, the paralyzed forepaw representation area was found in the ipsilateral motor cortex. At 8th month, the forepaw representation area was expanded. At 11th month, the forepaw representation area diminished and controlled the ipsilateral forepaw well.

**Figure 4:**
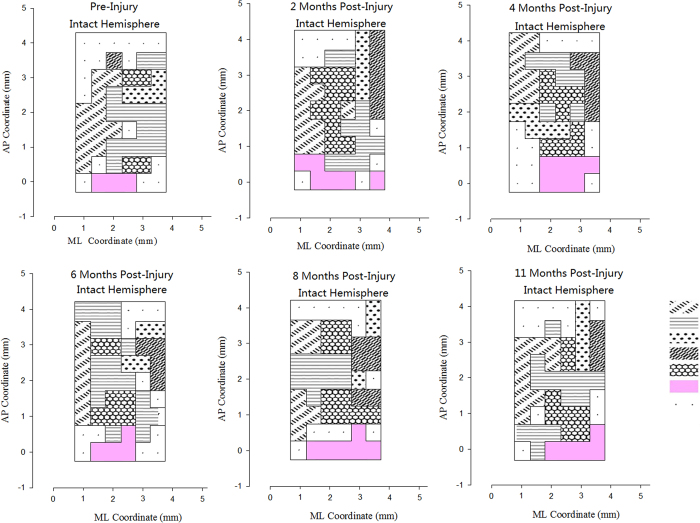
The primary motor cortex (M1) maps of intact hemispheres in group E (TBI_Control) from 2 to 11 months after traumatic brain injury. We could not found the paralyzed forepaw representation area in the intact hemisphere after the traumatic brain injury.

At the 6th month after surgery in Group F, movements of the left paralyzed forepaw could only be evoked when the left (Intact) M1 regions were stimulated. Meanwhile, stimulation of the left(Intact) M1 region could elicit movements of elbow, wrist, or paw exten- sion and adduction in the right healthy forelimb. The average responded points for left(paralyzed) forelimbs in left(intact) M1 region was 1.85 ± 0.75 at the 6th month after cross C7 transfer. At the 8th month, movements of the left forepaw could be evoked by stimulating left hemisphere of the M1 region and the cortical region expanded significantly, with average responded points of 6.33 ± 1.03 for left forepaw in M1 regions. At the 10th month, the left forepaw movement also could be evoked by stimulating the right M1 region, with the cortical region diminished, and the average responded points was 3.17 ± 1.17 at this time (Fig. 3). Meanwhile, in the control group (Group E), stimulation of the left M1 region could not elicit movements in the left forepaw (Fig. 4).

### Histological analysis

Weak staining for MAP-2, SYN and GAP-43 was observed in the control animals. At the end of 0.5 month, a dramatic increase in MAP-2, SYN and GAP-43 expression was detected in the residual ipsilesional hemisphere in group A (TBI_Control) and B (TBI_C7-Transfer) with no significant difference (p > 0.05). MAP-2 expression and GAP-43 expression subsequently decreased in both groups until 8 months. Moreover, increased MAP-2 and SYN appeared in the contralesional hemisphere in group B (TBI_C7-Transfer) at the end of 4 months and peaked at 8 months. Significant increased GAP-43 expression was also found in the contralesional hemisphere in group B (TBI_C7-Transfer) from 6 to 11 months. The intensity was significantly higher compared to group A (TBI_Control) (p < 0.05). At 11 months, the expression of MAP-2, SYN and GAP-43 subsequently decreased in the contralesional motor cortex but was still higher than group A (TBI_Control) (p < 0.05) (Table 1, Figs S3–S5 in Supplementary Material).

## Discussion

There has been wide discussion on the role of contralesional hemispheric compensation in functional recovery of the paralyzed limb after unilateral central nervous system injury, but no consensus has been achieved to date. In this study, a safe and effective approach was described to enhance the contralesional hemispheric compensation, which connects the contralesional hemisphere and paralyzed upper limb. Thus, this is an ideal model for investigating the role of the contralesional hemisphere in motor recovery after central nervous system injury. The model of cross-neck C7-C7 nerve transfer was designed for the following reasons: (1) a large body of evidence has been reported for the safety of sacrificing C7^9^,^10^,^16^,^17^; (2) the C7 nerve contains abundant nerve fibers, including both sensory and motor fibers; (3) the nerve crossing model has proven to substantially induce interhemisphere cortical reorganization^18^; and (4) most of the C7 fibers diffusely innervate extensor muscles, which remain much weaker than flexor muscles in stroke survivors in which the imbalance between flexors and extensors results in a closed and functionally useless hand^19^.

Rats lose connections between the injured hemisphere and contralateral paretic forelimb immediately after TBI. Through nerve transfer surgery, the paretic forelimb may gradually regain functional control by the contralesional hemisphere via two nerve crosses after regeneration of the peripheral C7-C7 pathway. In behavioral performance, TBI-only rats presented spontaneous motor recovery shortly after brain injury, although this recovery tended to be limited and the final motor functional outcome turned out to be very poor. In contrast, the behavioral performance in TBI rats with cross-neck C7-C7 nerve transfer continuously improved at 4 months and returned to the baseline score at the final observation. Thus, this further behavioral improvement seems exclusively attributed to the effect of cross-neck C7-C7 transfer.

Micro-PET imaging revealed functional brain areas associated with an improvement of the paretic forelimb. Activation of the residual ipsilesional hemisphere shortly after focal brain ablation indicated by micro-PET images at 0.5 month indicated the key mechanism for spontaneous functional recovery, ipsilesional reorganization. This activation in the residual ipsilesional hemisphere persisted throughout the 11 months period after brain injury in TBI-only rats. In addition to this, a new activated area in the contralesional hemisphere was found at 0.5 month after unilateral brain ablation in TBI-only rats. This may be explained by an up-regulation of brain excitability by over-use of the intact forelimb. Consistent with these results, stronger activation of the contralesional hemisphere in group B (TBI_C7-Transfer) was induced by a cross neck C7-C7 nerve transfer. In the longitudinal analysis, the residual ipsilesional hemisphere contributed to the spontaneous recovery in TBI-only rats. Reorganization of the contralesional hemisphere appeared to facilitate functional recovery in TBI rats with a cross neck C7-C7 nerve transfer. These results suggested that the adaptive plasticity in the contralesional hemisphere was newly formed after cross-neck C7-C7 transfer, which may have contributed to the significant motor recovery of the paralyzed forelimb.

In intracortical microstimulation experimental, the neural electrophysiology results showed that the paralyzed forepaw was controlled directly by the ipsilateral intact primary motor cortex at 6 months after cross neck C7-C7 nerve transfer. The representative area was expanded at 8 months and finial diminished at 11 months after the operation. The occurrence of the representative area of the paralyzed forepaw in the ipsilateral hemisphere suggested that completion of peripheral nerve regeneration helped to reestablish a new neural pathway between the brain and the paretic peripheral termination.

MAP-2 is an important structural protein not only for maintaining cytoskeletal integrity but also for neuronal growth, plasticity and regeneration and has been used to indicate cytoskeletal reorganization following brain injury^20^. Changes in MAP-2 in the brain predict related synaptic reorganization. SYN is a specific membrane protein located on the surface of synaptic vesicles and plays a vital role in physiological events, such as neurotransmitter release and synaptic plasticity^21^. An increase in SYN expression predicts the formation of a new synapse. Additionally, neuronal GAP43, as an indicator of synapse formation, is involved in mechanisms controlling path finding and branching of neurons during neural regeneration after injury. Up-regulated expression of ] MAP-2,SYN and GAP-43 were observed in the residual ipsilesional hemisphere at 0.5 month after brain injury, which represents rapid compensation via functional and structural reorganization. However, the expression of SYN, MAP-2 and GAP 43 in the residual ipsilesional hemisphere was subsequently decreased because only a small part of this initial connection remained established^22^. Similar to micro-PET outcomes, we also observed an increase in MAP-2, SYN and GPA43 expression in the contralesional hemisphere from 4 to 11 months, which was also synchronous with the successful regeneration of the peripheral cross-neck C7-C7 pathway. Completion of peripheral nerve regeneration helped to reestablish a new neural pathway between the brain and the paretic peripheral termination. It is likely that massive peripheral signal inputs contributed to the reorganization in the contralesional hemisphere and induced synaptogenesis.

According to functional and morphological evidence from neurophysiology test, micro-PET imaging and immunohistological analysis, spontaneous motor recovery could be mainly attributed to residual ipsilesional hemisphere reorganization and partially to contralesional hemisphere reorganization. However, further improvements in motor recovery in group B (TBI_C7-Transfer) compared to group A (TBI_Control) could be attributed to the surgical procedure and subsequent activation of the contralesional hemisphere.

The positive feedback effect that functioned through the newly formed double-crossed pathway was another possible contribution to better motor performance in group B (TBI_C7-Transfer). With the peripheral rearrangement, the contralesional hemisphere was more activated, thus facilitating motor recovery. It has been hypothesized that sensory input is important in the modulation of neuroplasticity during motor skill learning^23^. With an improvement in motor function, rats in group B (TBI_C7-Transfer) increasingly used their paretic forelimbs when rearing, exploring and eating. Movements with recovering muscles and joints, in turn, continuously delivered proprioceptive feedback to the brain and excited the contralesional hemisphere.

In conclusion, our preliminary studies revealed that cross neck C7-C7 transfer could largely strengthen contralesional hemisphere compensation at the chronic plateau stage of TBI rats. Thus, this procedure may pave the way for advancing the translation of neuroplasticity research toward clinical applications because major advances in the understanding of neuroplasticity have hardly yielded promising interventions thus far.

## Methods

### Animal model of C7 cross-transfer

All experiments were performed in accordance with relevant guidelines and regulations. All experimental protocols were approved by Institutional Review Board of Huashan Hospital.

For histological analysis, we divided 72 adult female SD rats into two groups: (Group A TBI_Control) 36 rats underwent a unilateral motor cortex ablation; and (Group B TBI_C7-Transfer) 36 rats underwent a unilateral motor cortex ablation and subsequently received a cross neck C7-C7 transfer 1 month later. In group A (TBI_Control) and B (TBI_C7-Transfer), the rats were divided into 6 subgroups (6 rats per subgroup) according to different time intervals at postoperative observation (0.5, 2, 4, 6, 8 and 11 months). The subgroups (11 months) in both groups were used for behavior observations.

For small animal PET investigation, we divided 12 adult female SD rats into two groups: (Group C TBI_Control) 6 rats underwent a unilateral motor cortex ablation; and (Group D TBI_C7-Transfer) 6 rats underwent a unilateral motor cortex ablation and subsequently received a cross neck C7-C7 transfer 1 month later.

For intracortical microstimulation, we divided 72 adult female SD rats into two groups: (Group E TBI_Control) 36 rats underwent a unilateral motor cortex ablation; and (Group F TBI_C7-Transfer) 36 rats underwent a unilateral motor cortex ablation and subsequently received a cross neck C7-C7 transfer 1 month later. In group E (TBI_Control) and F (TBI_C7-Transfer), the rats were divided into 6 subgroups (6 rats per subgroup) according to different time intervals at postoperative observation (pre, 2, 4, 6, 8 and 11 months).

### Unilateral motor cortex ablation

The anesthetized rats were placed in a prostrate position. After shaving and head skin disinfection, a midline incision was made to expose the skull from bregma to lambda and a 5-mm craniotomy (−0.5~4.5 mm anterior posterior, 0~5 mm mediolateral) was performed. Next, we resected the underlying left motor cortex using a surgical blade by a depth of 4 mm from the cortex surface in all rats in group A (TBI_Control) and C (TBI_Control) as previously described^24^,^25^.

### Cross neck C7-C7 transfer

**Figure 5:**
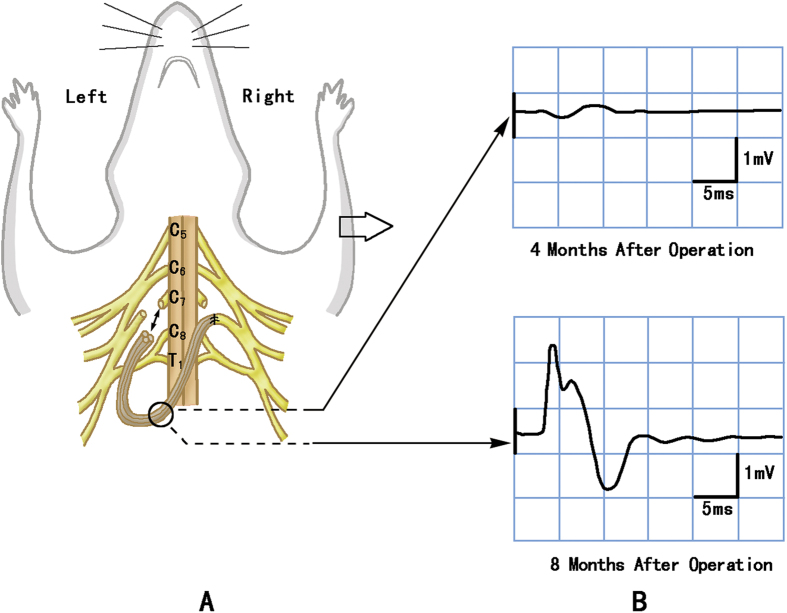
Schematic diagram of the cross neck C7-C7 transfer (****A****), action potential from the right triceps at different intervals after cross neck C7-C7 transfer (**B**). (**A**) Schematic showing that the distal cut end of the right (paralyzed forelimb side) C7 nerve was severed near the spine and connected with the proximal cut end of the left (healthy forelimb side) C7 nerve via nerve grafting. (**B**) Right triceps (paralyzed side) showed a small action potential while the nerve graft was stimulated at 4 months after TBI. The wave form appeared definite with 2 mV amplitude and 3 ms latency at 8 months.

For the rats in group B (TBI_C7-Transfer) and group D (TBI_C7-Transfer), the anesthesia and unilateral motor cortex ablation procedures were identical to those performed in group A (TBI_C7-Control). One month later, all of the rats were placed in a supine position; a cervical median incision of approximately 1.5 cm in length was made, starting from the suprasternal fossae, and a layered dissection was performed. We dragged the bilateral clavicles distally and exposed and confirmed the C7 nerve (including dorsal and ventral roots) on both sides. Next, bilateral C7 nerves were fully exposed and transected. The proximal cut end of left C7 nerve was connected with the distal cut end of the right C7 nerve, via 4 strands of the interpositional autograft sural nerve (Fig. 5A).

At each postoperative interval, electrical action potentials were recorded from the right triceps using a needle electrode while stimulating the nerve graft at the neck to confirm regeneration of the peripheral C7-C7 pathway (Fig. 5B).

### Behavioral observations

The skilled reaching task has been previously described for the assessment of forelimb coordination and fine digit motor control after brain injury^26^. All animals were trained 5 days per week, over 2-week sessions prior to surgery. During the training period, a strip of rubber band was used to fix the left forelimb with the ipsilateral hind limb, to prevent the rat from inserting the forelimb through the window to grasp food. Each training session consisted of reaching and grasping 20 pellets for 10 min each day using the right forelimb. The performance of each rat was then calculated using the following formula: success percentage = (number of pellets retrieved ÷ number of reach attempts) × 100. All testing sessions were video recorded for subsequent analysis.

The limb-use asymmetry test was performed to examine the functional impairment of forelimb preference during exploratory behavior^27^. The rat was placed in a transparent cylinder and video recorded for 3–10 min depending on the degree of movement maintained during the trial. Two mirrors were placed behind the glass cylinder at an angle of 90°. Several forelimb impairments during spontaneous exploration of the glass cylinder were recorded to determine the effect of the brain injury. The behaviors were analyzed during rearing and exploration along the wall using the left or right forelimb exclusively or simultaneous forelimbs: (a) first contact with the wall; (b) vertical and horizontal movements along the wall; and (c) sliding movements of each forelimb at the wall of the cylinder. Forelimb activity (FLA) = (first contact + horizontal + vertical) ÷ number of rearing. Sliding score (%) = sliding ÷ (first contact + horizontal + vertical) × 100.

### [F-18] FDG micro-PET scan

Rats were transiently anesthetized for approximately 1 min using halothane gas. Specific activity of 500 Ci/mmol FDG was prepared before the injection procedure. The rats were then injected i.v. with 0.5 m Ci FDG through the dorsal penile vein and then returned to the home cage. Then, we waited approximately 30 min for sufficient uptake of the [F-18] FDG. After the tracer uptake stage, the animal was placed in a spread legged prone position and scanned using the micro-PET R4 (Concorde Microsystems, Knoxville, TN, USA) under halothane gas anesthesia (5% induction and 1.5% for maintenance), which consists of a 15-cm-diameter ring of 96 position-sensitive ray scintillation detectors, which provides a 10.8-cm trans-axial and a 7.8-cm axial field of view with an intrinsic resolution 1.8 mm. All of the static acquisition was continued for 15 min in 3D mode. All imaging data were constructed using a max-posterior probability algorithm with a pixel size of 0.4 × 0.4 × 1.2 mm^3^ by a specified technician^28^.

Voxel-based statistical analyses were performed using SPM8 software: paired t-test for comparison between different time points in the same group, and multiple regressions for longitudinal analysis using scores obtained from the reaching task as covariates. The statistical threshold was set at *p* = 0.05 (FDR correction).

### Intracortical Microstimulation

At each intervals before and after opertaion (2, 4, 6, 8, 11month), the rat was placed in a special stereotaxic instrument (Jiangwan Type I-C, The Affiliated Instrument Factory of the Second Military Medical University, Shanghai, China). Then part of the left parietal skull bones was removed between 6 mm anterior to and 4 mm posterior to Bregma. It corresponds to Horsley–Clarke coordinates A9.0, L0, and from 0 to 4. 5 mm lateral to the midline. We used microelectrodes with tip impedances of 0.2–1.0 M for cortical stimulation. The cortical surface covering the area between 5 mm anterior to and 1 mmposterior to Bregma and from 0.5 to 4 mm lateral to the midline was tested to map motor representations in steps of 0.5 mm. The electrode was put to a depth of 1.8 mm below the cortical surface. According to the literature, this depth was found to correspond to layer V of the frontal cortex. The locations of penetration were examined by viewing the histology section. Mono-phasic cathodic pulse trains (train length 75 ms, pulse duration 0.25 ms at temporal frequency 200 Hz) were generated by a constant current stimulator (Model: U-ML180-OG-02A, MacLab, Powerlab, AD Instruments, Castle Hill, New South Wales, Australia), which were used to evoke movements of the forepaw or the body. The tested point in motor cortex which could evoke a visible movement of a forepaw was defined as a responded point.

The software of origin 6.1 (Origin Lab Corporation, Northhampton, MA) was used for data analyze. We drew the responded points in anterior posterior coordinate (AP) and medial lateral coordinate (ML) in the M1 region. The recorded responded points represented the distance from former top of head and sagittal suture to the stimulation location. Motor cortical maps were constructed by mapping the location in which electrical stimuli evoked a visible or palpable movement. The movement map of other body parts such as the mouth or vibrissae that were elicited in cortical are as adjacent to the forelimb are as were also constructed.The responded points numbers from stimulation of cortex for different body parts were counted.

### Histological analysis

After the behavioral test, all of the rats at 0.5, 2, 4, 6, 8 and 11 months were deeply anesthetized with sodium pentobarbital and perfused with a solution of 4% paraformaldehyde. Following cervical section, the brains were rapidly removed for fixation overnight in 4 °C paraformaldehyde (4% in PBS). After washing three times for 2 h in PBS (4 °C), the brains were incubated in 30% sucrose in PBS solution at 4 °C for 2–3 days until they sank. Subsequently, the brains were frozen in OCT on dry ice and stored at −80 °C. Coronal sections of 6 μm were prepared on positively charged glass microscope slides. Sections were dried at least 2 h at room temperature (RT) and then stored at −20 °C until immunostaining.

After the sections were fixed for approximately 15 minutes, 6-μm-thick sections were washed with 0.01 M PBS and then incubated in anti-MAP2 (polyclonal antibody, dilution at 1:200, from Santa Cruz, USA) or anti-Synaptophysin (polyclonal antibody, dilution at 1:400, from Santa Cruz, USA) or anti-GAP43(monoclonal antibody, dilution at 1:100, from Abcam, UK) at 37 °C for 2 h, followed by 4 °C overnight. Diaminobenzidine (DAB) was used as the chromogenic substrate with the LSAB Kit (Dako Corporation, Santa Barbara, CA).

Five areas (173 μm2/area) of each sample were microscopically examined and analyzed by an experienced neuropathologist. Images were captured using a charge-coupled device (CCD) camera and analyzed using Leica Qwin Advanced software (Leica, Germany). The average of the staining score was calculated by dividing the positive areas by the total area.

## Additional Information

**How to cite this article**: Hua, X.-Y. *et al.* Enhancement of Contralesional Motor Control Promotes Locomotor Recovery after Unilateral Brain Lesion. *Sci. Rep.* **6**, 18784; doi: 10.1038/srep18784 (2016).

## Supplementary Material

Supplementary Information

## Figures and Tables

**Table 1:** The expression of MAP-2 and Synaptophysin (SYN) in perilesional area and contralesional hemisphere in groups A and B rats at each interval after left sensorimotor cortex injury (%, 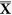 ± S).

Time (month)	Group A		Group B	
	perilesional area	contralesional hemisphere	perilesional area	contralesional hemisphere
pre-TBI	4.67 ± 0.19	4.44 ± 0.23	4.67 ± 0.19	4.64 ± 0.33
0.5	7.24 ± 0.63*	5.78 ± 0.46	8.09 ± 0.49*	5.35 ± 0.46^*^
2	5.82 ± 0.33	5.61 ± 0.31	5.56 ± 0.23^*^	5.41 ± 0.17^*^
4	5.07 ± 0.46	4.75 ± 0.19	4.87 ± 0.46	5.25 ± 0.19^*^
6	4.8 ± 0.27	4.44 ± 0.21	4.91 ± 0.37	7.74 ± 0.21^*^
8	4.7 ± 0.21	4.61 ± 0.23	4.83 ± 0.41	9.78 ± 0.76^*^
11	4.48 ± 0.32	4.52 ± 0.42	4.78 ± 0.22	7.06 ± 0.42^*^
**SYN Expression**				
pre-TBI	3.9 ± 0.21	4.05 ± 0.27	3.9 ± 0.21	3.86 ± 0.18
0.5	4.71 ± 0.24	4.79 ± 0.44	4.82 ± 0.39	4.68 ± 0.44
2	4.2 ± 0.20	4.5 ± 0.33	4.31 ± 0.20	4.5 ± 0.33
4	3.65 ± 0.23	3.75 ± 0.33	3.99 ± 0.24	5.66 ± 0.33^*^
6	3.6 ± 0.21	3.48 ± 0.21	4.16 ± 0.32	6.68 ± 0.52^*^
8	3.5 ± 0.17	3.55 ± 0.19	4.24 ± 0.27	7.83 ± 0.81^*^
11	3.48 ± 0.19	3.45 ± 0.23	4.18 ± 0.20	6.45 ± 0.49^*^
**GAP-43 Expression**				
pre-TBI	3.5 ± 1.04	2.83 ± 1.17	4 ± 0.89	4.33 ± 1.37
0.5	8.17 ± 1.17	3.67 ± 1.21^*^	6.17 ± 1.47	5.17 ± 1.72
2	7.83 ± 2.04	3.33 ± 1.37^*^	6.5 ± 1.05	5.17 ± 0.33
4	5 ± 0.89	4 ± 2.1	5.17 ± 0.98	6.5 ± 1.05
6	5.33 ± 1.03	3.83 ± 2.79	6.32 ± 1.17	10.67 ± 1.63*
8	5.5 ± 1.38	5 ± 1.67	5.17 ± 1.17	10.17 ± 1.32*
11	5.17 ± 1.17	4.67 ± 1.37	5.67 ± 1.63	8.67 ± 1.51*

^*^Indicating significant differences between perilesional area and contralesional hemisphere in the group (*p* < 0.05).
