# Supplementary material for: Enhancement of Contralesional Motor Control Promotes Locomotor Recovery after Unilateral Brain Lesion
Source: Sci Rep. 2016 Jan 6;6:18784. doi: 10.1038/srep18784 (PMC4702126; doi:10.1038/srep18784)
Supplement: Supplementary Information [file srep18784-s1.doc]

**Enhancement of Contralesional Motor Control Promotes Locomotor Recovery after Unilateral Brain Lesion**

Xu-Yun Hua^1*^, Yan-Qun Qiu^1,2,*^, Meng Wang^3*^, Mou-Xiong Zheng^1^, Tie Li^1^ ,Yun-Dong Shen^1^ , Su Jiang^1^, Jian-Guang Xu^1^, Yu-Dong Gu^1^,JoeZ.Tsien^4,5^ and Wen-Dong Xu^1,2,6,#^

^1^Department of Hand Surgery, Huashan Hospital, Shanghai Medical College, Fudan University, Shanghai, China

^2^Department of Hand and Upper Extremity Surgery, Jing’an District Central Hospital, Shanghai, China

^3^Hand-Foot Surgery Department, Shandong Provincial Hospital, Shandong, China

^4^Brain and Behavior Discovery Institute and Department of Neurology, Medical College of Georgia, Georgia Health Sciences University, Augusta, GA 30907, USA.

^5^Yunnan BanNa Primate Model Research Center, BanNa Biomedical Research Institute, Xishuangbanna, Yunnan, China

^6^State Key Laboratory of Medical Neurobiology, Fudan University, Shanghai, China

^*^**These authors contribute equally to this work**


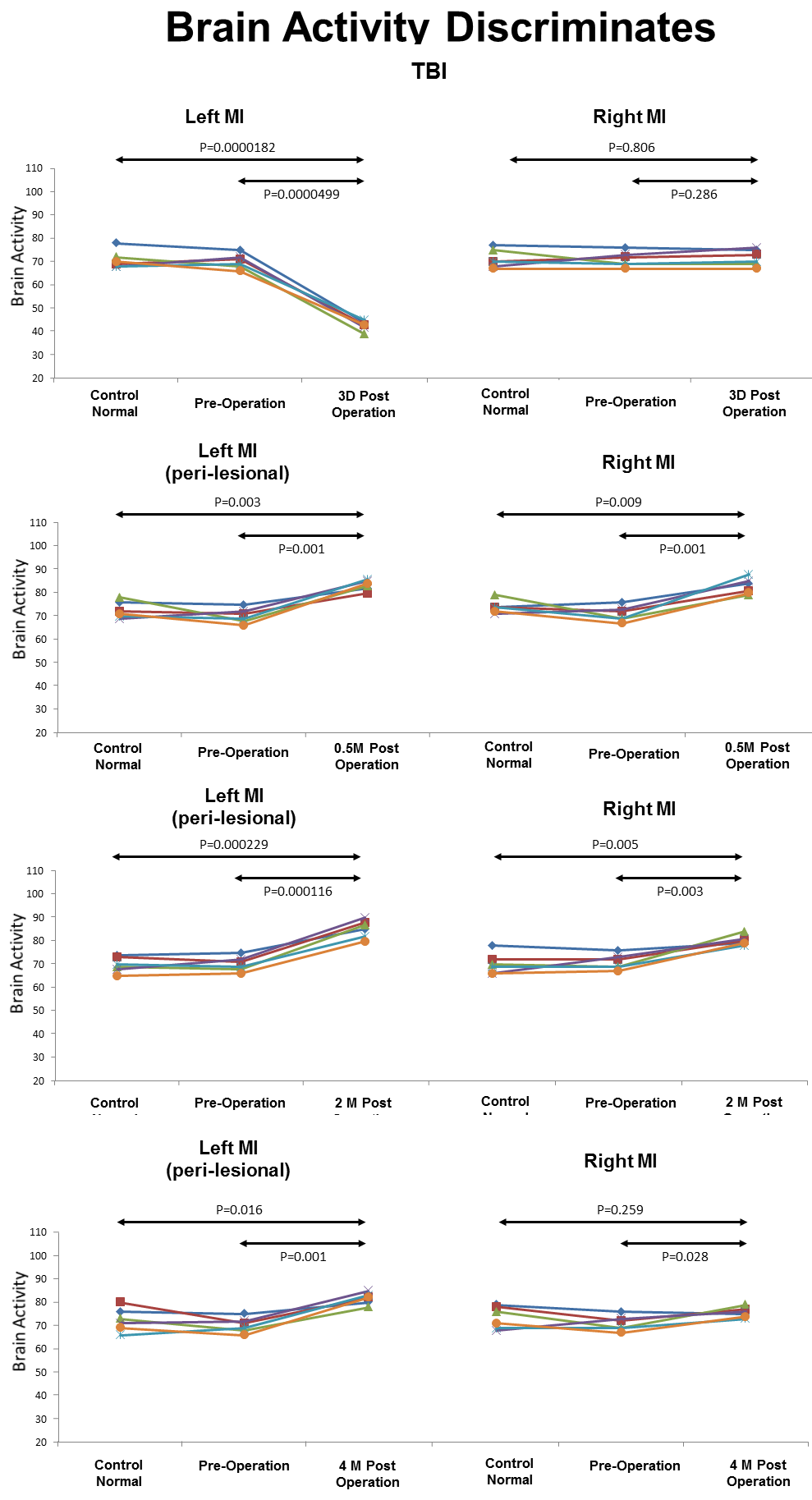


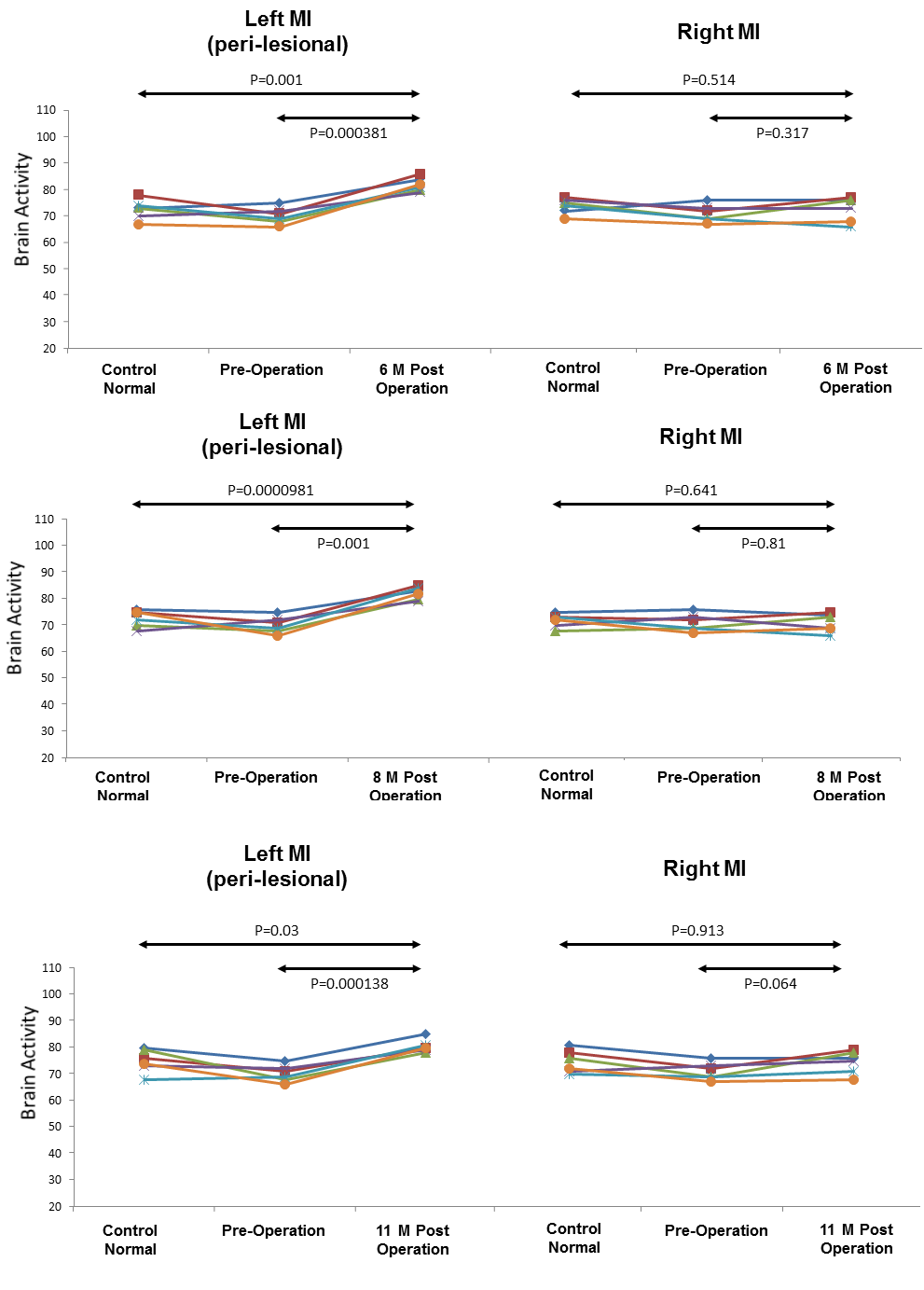


**Figure S1: Brain activity discriminates of TBI rats’ Micro-PET results:**

The brain activity for the comparison between control normal, pre-operation and different post-operative intervals and for longitudinal analysis, indicated by the value of glucose metabolism in micro-PET paired t-test.


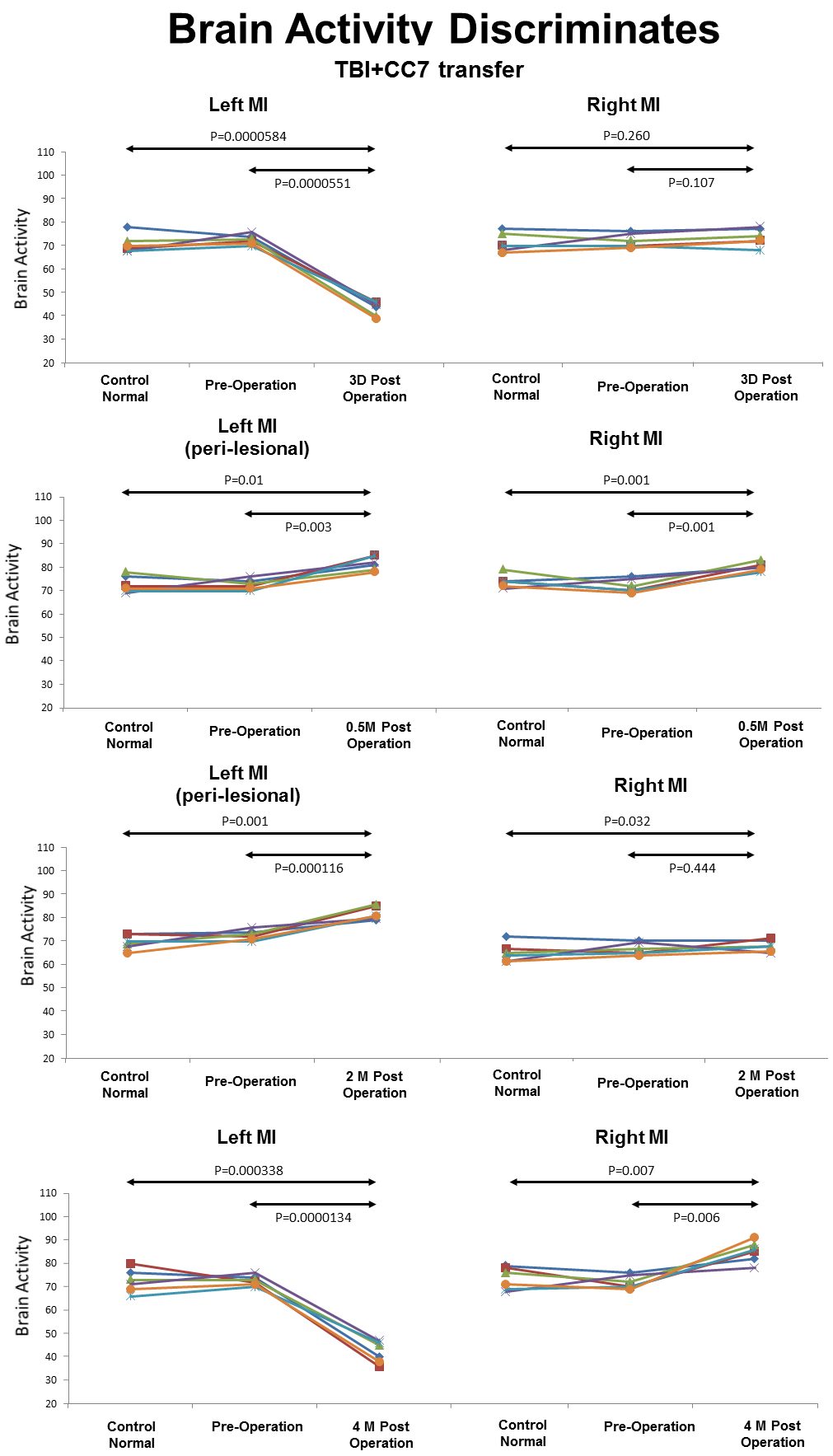


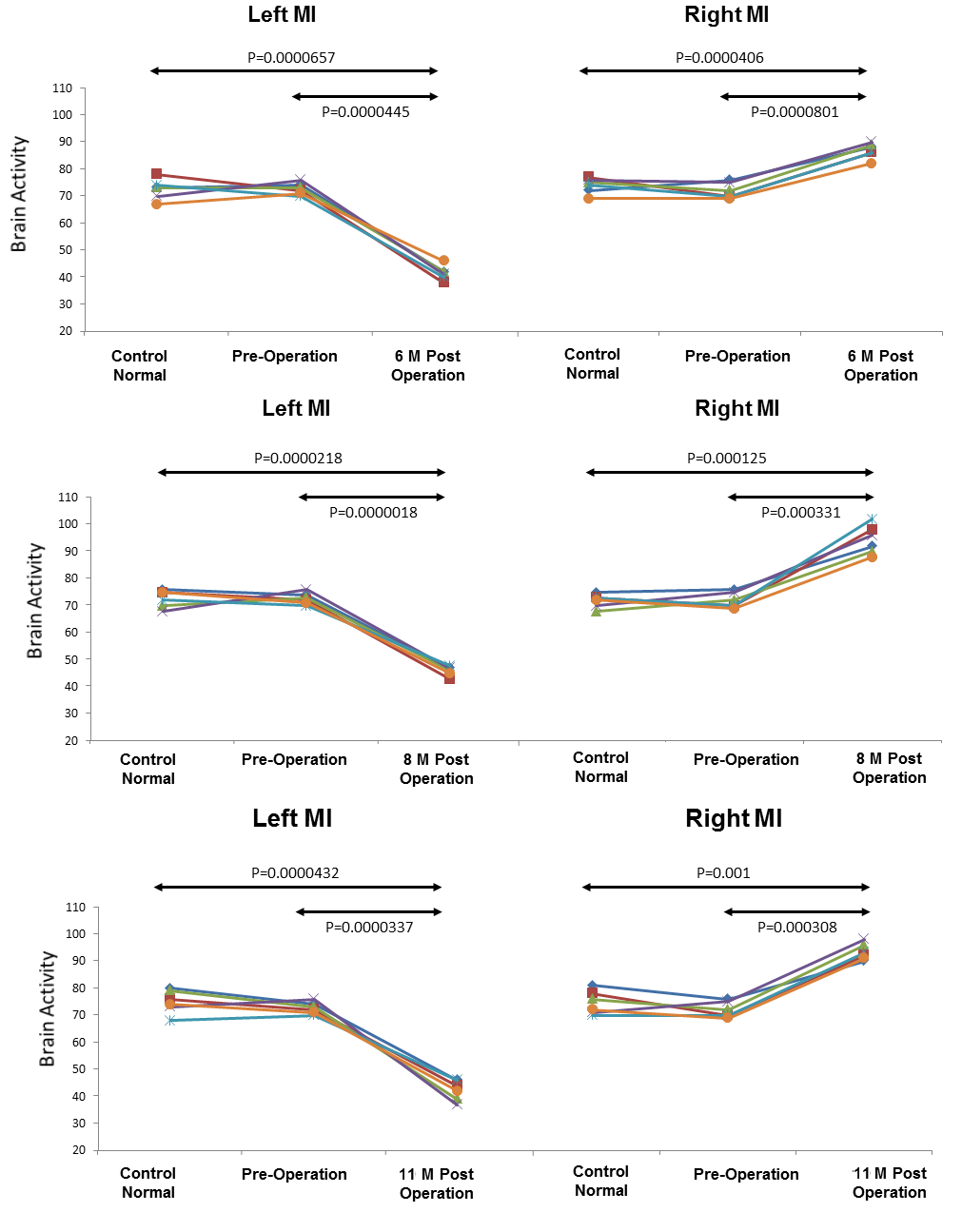


**Figure S2: Brain activity discriminates of TBI+CC7transfer rats’ Micro-PET results:**

The brain activity for the comparison between control normal, pre-operation and different post-operative intervals and for longitudinal analysis, indicated by the value of glucose metabolism in micro-PET paired t-tes


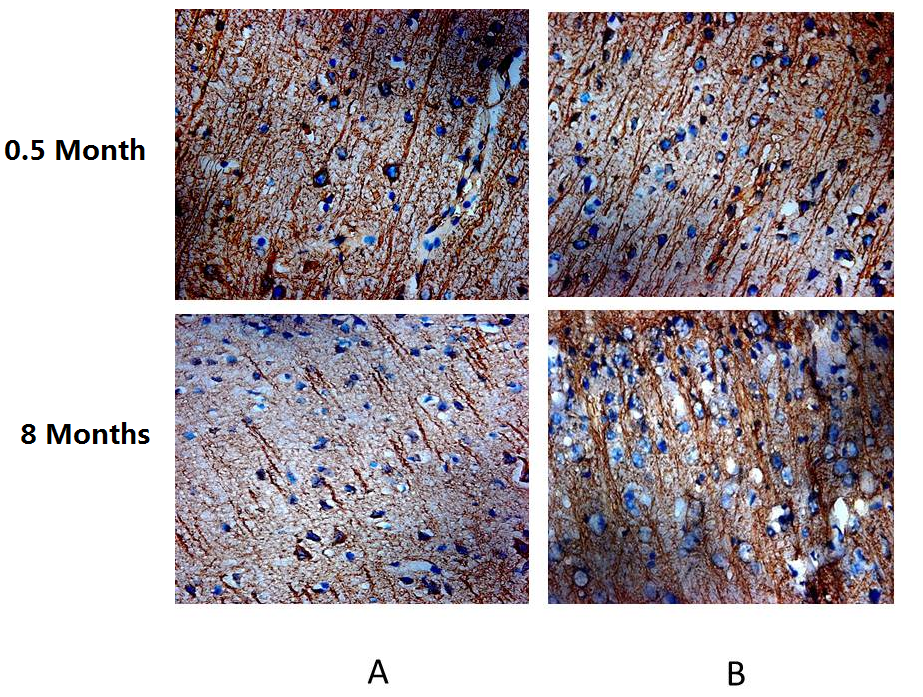


**Fig. S3: MAP-2 expression alternations in two groups at different time points after operation.**

A dramatic increase of MAP-2 expression was detected in the peri-lesion area in group A and B at 0.5 months. At 8 months, increased expression of MAP-2 appeared in the contralateral healthy hemisphere in group B, significantly higher than that of group A (p<0.05)


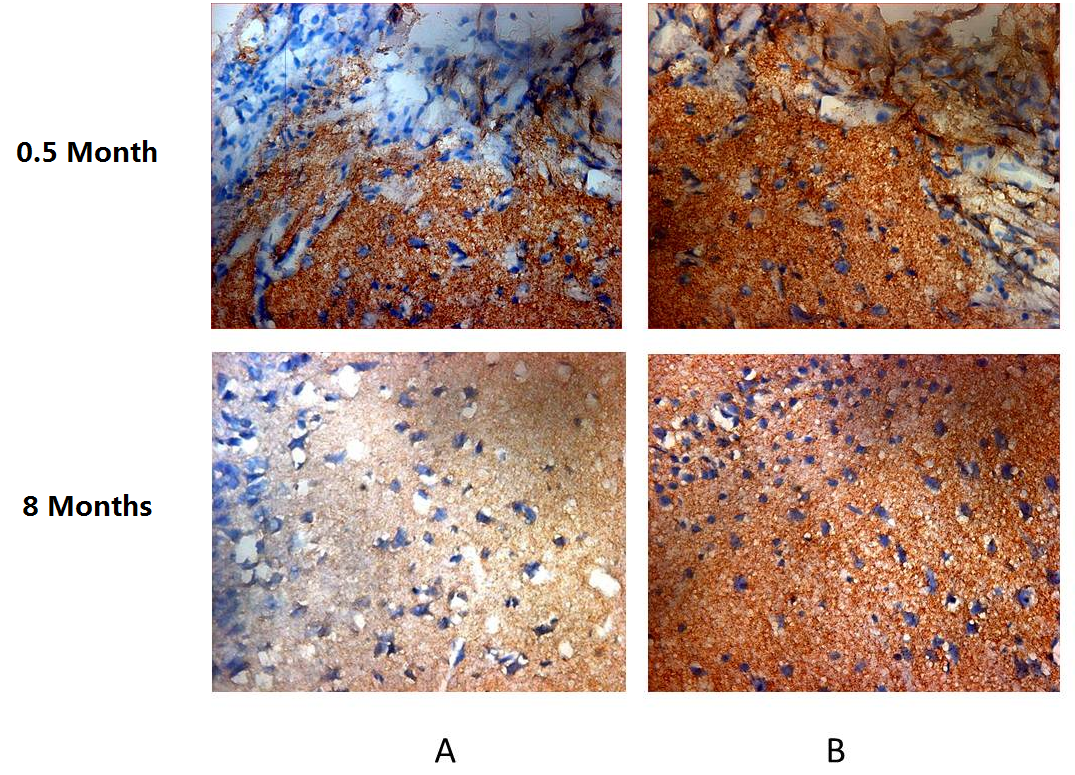


**Fig. S4: SYN expression alternations in two groups at different time points after operation.**

SYN expression increased in the peri-lesion areas and reached the peak at 0.5 month in group A and B, with no difference (p> 0.05). SYN of contralateral hemisphere in group B was significantly higher than that of group A at 8 months (p<0.05).


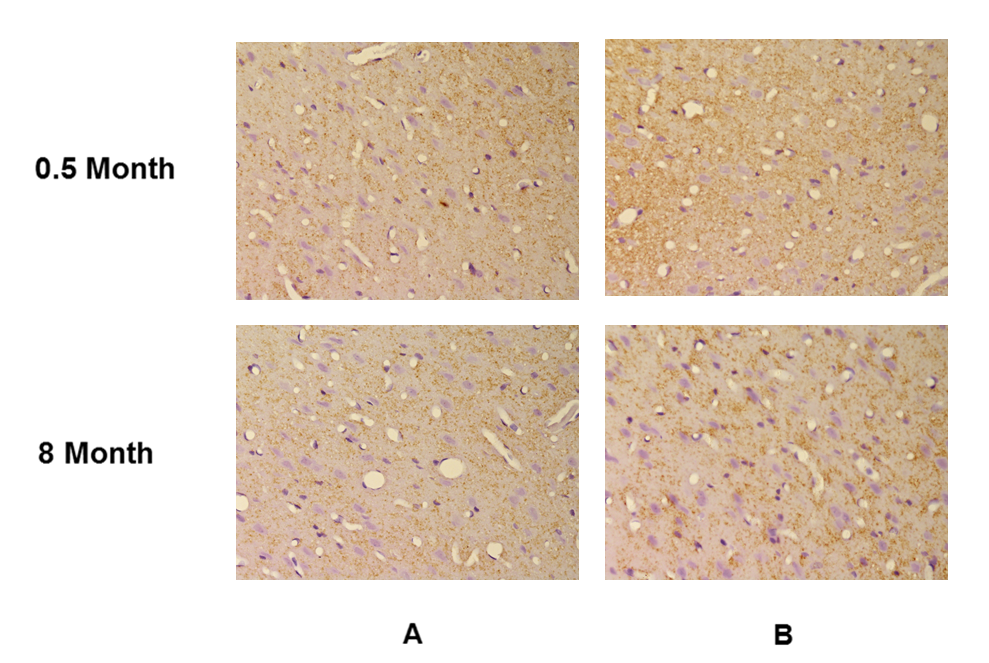


**Fig. S5: GAP-43 expression alternations in two groups at different time points after operation.**

GAP-43 expression increased in the peri-lesion areas at 0.5 month in group A and B, with no difference (p> 0.05). GAP-43 expression of contralateral hemisphere in group B was significantly higher than that of group A from 6 to 11 months (p<0.05).
